# Moisture Migration Behavior During Paddy Rice Drying Under Multi-Factor Stress and Its Effects on Starch, Protein, and Antioxidant Enzyme Activities

**DOI:** 10.3390/molecules31213908

**Published:** 2026-10-29

**Authors:** Simin Xing, Zimu Lin, Xianglan Gao, Xu Zhao, Guohui Liu, Yicen Chen, Jia Li, Lingzhi Wei, Dehua Wang

**Affiliations:** 1 Liaoning Grain Science Research Institute, Shenyang 110032, China; xingsimin024@126.com (S.X.);; 2 National Engineering Research Centre for Grain-Corn Drying, Shenyang 110032, China

**Keywords:** paddy rice drying, moisture migration, nutritional quality, drying models, antioxidant enzymes

## Abstract

To investigate the effects of drying conditions on moisture migration and quality changes in paddy rice, freshly harvested grain from northeastern China in autumn 2025 was subjected to 30 thin-layer drying treatments at 30–50 °C, 20–30% relative humidity, and 3–4 m/s air velocity. Three-dimensional interpolation surfaces of mean water-loss rate were constructed, and apparent effective moisture diffusivity was analyzed to characterize moisture migration. Six models—Newton, Page, Henderson–Pabis, Logarithmic, Wang–Singh, and Midilli—were fitted to evaluate their potential for process applications. Changes in dry basis protein and amylose contents and in peroxidase (POD) and superoxide dismutase (SOD) activities before and after drying were also examined. The results showed that temperature was the domi nant factor affecting the drying process. When the temperature increased from 30 to 50 °C, the mean drying time decreased from 125.00 to 48.33 min, and the mean water-loss rate increased from 0.204 to 0.662 g/min. The water-loss-rate surfaces rose along the direction of increasing temperature, with small fluctuations associated with changes in relative humidity and air velocity. The mean bed-scale apparent effective moisture diffusivity (Deff*) increased from 1.572 × 10^−8^ to 3.843 × 10^−8^ m^2^/s. Among the six drying models, Midilli provided the best in-sample fit, with an R^2^ of 0.9988 and an *RMSE* of 0.0082. It also generally yielded lower Akaike information criterion (*AIC*) values, suggesting its potential for detailed characterization of drying kinetics and process-oriented applications. The Logarithmic model ranked second, while the Page model also offered practical advantages owing to its simplicity. Temperature had a significant main effect on protein content (*p* = 0.011), with the lowest mean at 50 °C (7.900 g/100 g). The main effect of air velocity on amylose content was significant, but it did not remain significant in the HC3 sensitivity analysis. None of the three drying factors had a significant main effect on POD or SOD activity. The Pearson correlations of protein and amylose with SOD were significant; after Holm correction, only the protein–SOD negative correlation remained significant. Higher temperatures improved drying efficiency within the tested range, whereas quality indicators responded differently. Based on the observed changes in drying efficiency and quality indicators, 35–40 °C can be regarded as a candidate temperature range that balances dehydration efficiency and quality preservation, providing a reference for subsequent process optimization. These findings provide physiological and biochemical information to support the coordinated evaluation of moisture migration and quality during thin-layer paddy drying and the future development of intelligent drying-control models.

## 1. Introduction

Rice (*Oryza sativa* L.) is an important annual cereal crop of the Poaceae family and is widely cultivated in tropical, subtropical, and temperate regions under irrigated lowland, rainfed lowland, and upland production systems. The growth and development of rice are closely related to temperature, water supply, and light conditions, and there are obvious differences in cultivation systems and harvest seasons among different ecological zones. As one of the major staple food crops worldwide, rice plays an important role in dietary supply and food security assurance [1,2,3]. According to the latest statistics from the Food and Agriculture Organization of the United Nations (FAO), the global paddy rice production in 2024 was approximately 820 million t, of which about 734 million t was produced in Asia, making it the major global paddy rice production region [4,5]. China is also a major country in paddy rice production and consumption. According to data from the National Bureau of Statistics of China, the sown area of paddy rice in 2025 was 29.0122 million hm^2^, with a total production of 209.041 million t [6]. Given the enormous scale of paddy rice production, postharvest drying is a key step for quality preservation and safe storage, and has prominent economic and industrial significance [7,8,9].

Hot-air drying is widely used for paddy because it is simple to operate, adaptable, and capable of handling large quantities of grain. Its application nevertheless requires care, as paddy is sensitive to heat. During drying, heat is transferred to the grain, moisture diffuses from the interior, and water evaporates at the surface; these processes occur alongside changes in grain tissues. On the one hand, parameters such as temperature, relative humidity, and air velocity determine the moisture migration rate; on the other hand, they may also alter major nutritional components such as proteins and starch, and further change the activities of antioxidant enzymes such as peroxidase (POD) and superoxide dismutase (SOD) by affecting reactive oxygen species metabolism and antioxidant defense processes [10,11]. Drying performance thus needs to be assessed through both water removal and grain quality. Focusing on the dual objectives of dehydration efficiency and quality preservation, existing studies have mainly concentrated on moisture migration and diffusion characteristics, thin-layer drying model fitting and comparison, as well as the effects of drying conditions on nutritional quality and enzyme activities.

Moisture diffusion within the grain is a key determinant of drying efficiency. Both temperature and initial moisture content affect effective moisture diffusivity: even at a fixed temperature, samples with different initial moisture contents can exhibit different diffusion behavior [12]. Models have also addressed the scales at which transport occurs. Motazedian et al. [13] established a local non-equilibrium heat and moisture transfer model at the kernel and pore scales to describe the temperature and moisture distributions in individual kernels and kernel assemblies. At the bed scale, Li B et al. [14] investigated changes in the porosity of a flowing paddy layer by combining experiments with numerical simulations. They considered grain flow rate, moisture content, air velocity, and layer thickness, providing a basis for examining the relationship between bed structure and airflow. Starting from heat and mass transfer and motion behavior at the particle scale, Prakotmak et al. [15] used a coupled computational fluid dynamics–discrete element method (CFD–DEM) to analyze the heat transfer and motion characteristics of paddy rice particles in a rotary drum, further enriching the understanding of complex drying processes. Drying schedules offer another means of influencing moisture redistribution. Garcia-Llobodanin et al. [16] showed that adequate tempering can complete paddy drying without reducing the head rice yield, but it alters the pasting properties and sensory attributes, and the sensitivity to drying conditions differs among varieties. Yuan et al. [17] combined variable-humidity drying experiments with heat–moisture transfer and stress–strain modeling, finding that staged dehumidification could limit stress accumulation and endosperm fissuring. In a closed-loop heat-pump system, Huang et al. [18] reported effects of air humidity on system performance, drying progress, and grain quality. These studies address different materials, bed configurations, and air conditions, all of which matter when interpreting transport parameters. Comparisons made with the same material and apparatus can help clarify how combinations of temperature, relative humidity, and air velocity affect dehydration behavior.

Mathematical modeling and comparison of thin-layer paddy drying have advanced considerably in recent years. Drying models express the temporal evolution of moisture ratio in a form that permits quantitative comparison. The field has progressed from simple empirical equations to modified and multiparameter models and, more recently, to data-driven prediction. Early Lewis or Newton formulations describe moisture-ratio decay using a first-order exponential function [19]. Page introduced an additional exponent, n, to better represent nonlinear behavior during falling-rate drying [20]. The Henderson–Pabis model extends the exponential formulation by including a scaling coefficient [21], whereas the Midilli model combines exponential and linear terms and has fitted single-layer drying data well for several agricultural products [22]. In paddy drying, Ozukum et al. [23] designed response-surface experiments combining different bed thicknesses, power levels, air flow rates, and exposure times, analyzed the variation in the effective moisture diffusivity, and found that the Page model could predict the moisture ratio well. Hasibuan et al. [24] compared seven existing drying kinetic models based on fluidized bed dryer experiments, and determined, using six statistical parameters, that the Verma drying kinetic model was the most suitable model for paddy rice under all the given temperatures (50 °C, 60 °C, and 70 °C) in the fluidized bed dryer. For infrared drying, studies have also validated the applicability of empirical models to novel drying methods through multi-factor experiments and model comparison [25]. Jin et al. [26] jointly considered temperature, relative humidity, initial moisture content, and air velocity and related accumulated temperature to paddy quality, providing a basis for controlling several operating parameters. Response-surface and neural-network approaches have subsequently extended the focus from fitting curves under individual conditions to predicting process behavior and quality across combinations of factors [27]. Although a broad range of kinetic models and optimization approaches is available, many comparisons remain restricted to individual drying methods or limited operating ranges [28,29]. The applicability of different models under combined conditions of temperature, relative humidity, and air velocity still needs to be further compared within a unified experimental system, and the sensitivity of the relevant model parameters and their relationship with quality changes also deserve further investigation.

The effects of drying conditions on nutritional quality and enzyme activities are equally important. Assessing paddy drying requires attention to milling performance, nutritional composition, and physiological activity as well as drying efficiency. Variable-temperature drying can affect amylose content and milling and eating quality, and temperature may also influence measured protein content. However, the improvement in drying efficiency and the improvement in different quality indicators are not always synchronized, and different evaluation indicators respond differently to the same process [30]. The heating schedule shortened drying time and raised the taste score, but lowered head rice yield and amylose content. Liu et al. [31] used glass-transition theory to examine staged drying and found differences in drying rate and milling quality between processes. In contrast, changes in protein and amylose contents were similar between variable-temperature treatments in the rubbery and glassy states. Such results show why nutrient contents and milling and eating quality need to be evaluated separately for the drying conditions under study. Enzyme measurements offer an additional view of the grain’s response to drying. Huang et al. [32] reported that drying temperature affected reactive oxygen species accumulation, antioxidant enzyme metabolism, and vigor in rice seeds with different initial moisture contents. Antioxidant enzyme activities can therefore complement other measurements of physiological responses [33,34]. However, evidence obtained from seed-vigor studies needs to be considered in terms of the material and endpoints examined before it is applied to rice intended for food. Although these studies suggest that enzyme responses may accompany changes in quality during drying, few have systematically examined drying kinetics, nutritional composition, and antioxidant enzyme activities together. How moisture migration relates to these nutritional and enzymatic changes remains to be clarified.

Previous studies have thoroughly investigated the drying kinetics of paddy rice, the performance of empirical models, and individual quality responses; however, differences in materials, equipment, and operating conditions make it more difficult to compare results across studies. From the perspective of postharvest handling practices for paddy rice, the key issues in paddy rice drying research lie not only in improving drying efficiency, but also in clarifying the changing patterns of moisture migration behavior and quality indicators under the combined effects of temperature, relative humidity, and air velocity, and in establishing comparable experimental evidence between kinetic parameters and quality responses. Therefore, it is necessary to simultaneously evaluate dehydration behavior and changes in protein, amylose, POD, and SOD within the same temperature–humidity–air velocity combination system. Accordingly, this study investigated thin-layer hot-air drying of paddy rice under different combinations of temperature, relative humidity, and air velocity. Continuous weighing data were used to analyze mass loss, apparent moisture ratio, drying rate, and apparent effective moisture diffusivity and to evaluate the fit of mathematical models. These analyses were combined with measurements of protein and amylose contents and POD and SOD activities at the drying endpoint to characterize responses in nutritional quality and the antioxidant enzyme system. The aim was to elucidate the synergistic effects of different drying factors on moisture migration and quality changes in paddy rice, thereby providing comprehensive data support and a theoretical basis for process optimization and quality control in paddy rice drying.

## 2. Results

### 2.1. Moisture Migration During Thin-Layer Drying

The mass ratio (*Mr*) and apparent moisture ratio (MR^*^) under different drying temperatures, relative humidities, and air velocities are shown in Figure 1. Panels (a), (b), and (c) compare temperature, humidity, and air-velocity conditions, respectively, and the insets show the corresponding final *Mr*. The overall profiles indicate that drying did not proceed at a constant rate throughout the process: mass decreased relatively rapidly at the beginning and more slowly toward the end. Li et al. [35] also reported falling-rate drying behavior in paddy rice, consistent with the initial evaporation of free water at the grain surface followed by internal moisture diffusion becoming increasingly rate-limiting. As drying progresses, resistance to moisture migration from the grain interior to the surface increases, resulting in a gradual decline in the water-loss rate [36].

Figure 1a compares changes in *Mr* and MR^*^ at different temperatures under a relative humidity of 20% and an air velocity of 3 m/s. Higher temperatures produced a faster decline in both variables, consistent with enhanced heat transfer and evaporation. The decrease in *Mr* was slowest at 30 °C, whereas samples dried at 50 °C lost mass over a much shorter period. The more rapid decline in MR^*^ at higher temperatures indicates a stronger driving force for moisture transport. The profiles were broadly consistent with falling-rate drying, during which internal moisture transport becomes increasingly limiting relative to surface evaporation. Although increasing temperature can accelerate drying, it may also increase internal moisture gradients and associated stresses in the grain.

Figure 1b shows the changes in *Mr* and MR* with drying time at different relative humidity levels, a drying temperature of 30 °C, and an air velocity of 3 m/s. *Mr* declined most rapidly at 20% RH and most slowly at 30% RH. Lower air humidity increases the water-vapor pressure difference between the grain surface and the drying air, thereby increasing the driving force for evaporation. Differences in MR^*^ were comparatively small, suggesting that relative humidity primarily altered drying rate within the investigated range without substantially changing the overall shape of the moisture-migration profiles.

Figure 1c shows the variations in *Mr* and MR* at air velocities of 3 m/s and 4 m/s under a drying temperature of 30 °C and a relative humidity of 20%. Under these conditions, both *Mr* and MR* exhibited a rapid decrease followed by a gradual slowdown. The limited improvement in drying at the higher air velocity may be attributable to a thinner boundary layer at the grain surface and enhanced convective mass transfer. The curves nevertheless remained close, with a much smaller separation than that between temperature treatments. Under these conditions, raising air velocity from 3 to 4 m/s, therefore, offered only a limited improvement in drying.

### 2.2. Combined Effects of Temperature, Relative Humidity, and Air Velocity on Water-Loss Rate

Figure 2 shows the interpolated mean water-loss rates derived from the 30 experimental datasets, with separate surfaces for air velocities of 3 and 4 m/s. Black points mark the measured values, while the surface colors and base contours depict the same interpolation. At both air velocities, the surfaces rise mainly along the temperature axis. Changes within the 20–30% RH range are smaller, although some fluctuations occur at individual temperatures, and rates are generally higher at 20% than at 30% RH. The surface at 4 m/s appears smoother than that at 3 m/s over the conditions examined. Previous studies have found that humidity is related to moisture transport under low-temperature paddy drying conditions; however, their different drying operating conditions preclude a direct quantitative comparison [37]. In line with Section 2.1, these patterns indicate that temperature should receive priority when controlling thin-layer hot-air drying. Relative humidity may offer an additional means of moderating drying conditions and potential quality risks.

### 2.3. Effects of Temperature on Drying Time and Mean Water-Loss Rate

Since temperature played a more dominant role in the experiments, the drying characteristics of paddy under different temperature conditions were further analyzed. Table 1 summarizes the mean drying time, water-loss, and mean water-loss rate at each drying temperature. Mean drying time decreased from 125.00 min at 30 °C to 101.67 and 73.33 min at 35 and 40 °C, respectively, and then to 51.67 and 48.33 min at 45 and 50 °C. The reduction was particularly pronounced over the 30–45 °C range.

Figure 3 presents the trends of average drying duration and average moisture loss rate with temperature, intuitively showing that increasing temperature has a pronounced positive effect on paddy drying efficiency. The error bars in the figure represent the degree of dispersion among different humidity and air velocity combinations. Mean water-loss rate increased from 0.204 g/min at 30 °C to 0.662 g/min at 50 °C, corresponding to an approximately 3.25-fold increase based on the unrounded treatment means. The shorter drying times at higher temperatures are qualitatively consistent with the findings of Zhang et al. [38] on paddy rice drying. However, Pereira et al. [39] further demonstrated that modifying the drying schedule can alter the effective drying time, highlighting that temperature is not the sole determinant of process performance.

### 2.4. Apparent Effective Moisture Diffusivity and Activation Energy

Table 2 summarizes the apparent effective moisture diffusivity (${D_{\mathrm{eff}}}^{*}$) values calculated using Equations (2), (5), and (6), together with the corresponding *R*^2^ values, for the 30 paddy rice treatments. The ${D_{\mathrm{eff}}}^{*}$ values ranged from 1.366 × 10^−8^ m^2^/s to 4.438 × 10^−8^ m^2^/s, and the *R*^2^ values ranged from 0.9001 to 0.9969. The last two columns of Table 2 present the mean ${D_{\mathrm{eff}}}^{*}$ values and the corresponding mean *R*^2^ values of the log-linear regression for the six combinations of relative humidity and air velocity at each temperature. ${D_{\mathrm{eff}}}^{*}$ increased with temperature, from 1.572 × 10^−8^ m^2^/s at 30 °C to 2.565 × 10^−8^ m^2^/s at 40 °C and 3.843 × 10^−8^ m^2^/s at 50 °C. The maximum value, 4.438 × 10^−8^ m^2^/s, occurred at 50 °C, 25% RH, and 4 m/s. All 30 linear regressions yielded reported *R*^2^ values of at least 0.9000, indicating that the fitted relationships described the transformed data reasonably well.

To further compare and analyze the differences in ${D_{\mathrm{eff}}}^{*}$ under different drying conditions, a three-factor factorial analysis was performed based on the results in Table 2 [40]. To reduce the influence of temperature-related variance in ${D_{\mathrm{eff}}}^{*}$ values, ${{\ln(D}_{\mathrm{eff}}}^{*})$ was used for the three-factor analysis, and the results are shown in Figure 4. Figure 4 presents the partitioning of the corrected total variation in ${{\ln(D}_{\mathrm{eff}}}^{*})$ into three main effects, three two-way interaction effects, and residual variation. The main effect of temperature accounted for 91.50% of the corrected total variation in ${{\ln(D}_{\mathrm{eff}}}^{*})$, indicating its dominant contribution among the investigated factors. Air velocity and relative humidity contributed 3.06% and 0.32%, respectively. The contributions of the temperature × air velocity, temperature × relative humidity, and relative humidity × air velocity interactions were 1.34%, 1.09%, and 0.70%, respectively. The remaining 1.99% represented residual variation, encompassing the unmodeled three-way interaction and experimental error that could not be separately estimated. None of the three two-factor interaction terms in the ${{\ln(D}_{\mathrm{eff}}}^{*})$ model reached a significant level; therefore, the contribution rates of the interaction terms are interpreted descriptively only.

Figure 5 illustrates the variations in apparent effective moisture diffusivity ${D_{\mathrm{eff}}}^{*}$. Specifically, Figure 5A shows the effect of temperature on ${D_{\mathrm{eff}}}^{*}$, whereas Figure 5B illustrates the interaction between temperature and air velocity. As shown in Figure 5A, ${D_{\mathrm{eff}}}^{*}$ increased markedly with increasing temperature. The figure also illustrates the variability among the six treatment combinations at each temperature level. Temperature groups sharing the same letter were not significantly different from each other. The interaction effect of temperature × air velocity on the apparent effective moisture diffusivity is shown in Figure 5B. At each temperature, the mean ${D_{\mathrm{eff}}}^{*}$ at 4 m/s was higher than that at 3 m/s, and the difference between them was greatest at 50 °C. These results indicate that, under the factor levels and bed conditions used in this experiment, the logarithmic variation in apparent effective moisture diffusivity was mainly associated with temperature changes [41,42].

The Arrhenius relationship was fitted using the mean diffusivity at each of the five temperatures. The regression of ${{\ln(D}_{\mathrm{eff}}}^{*})$ against reciprocal absolute temperature yielded *R*^2^ = 0.9815. The apparent activation energy (Ea) calculated using Equation (7) was 38.96 kJ/mol. In this study, ${D_{\mathrm{eff}}}^{*}$ was calculated using half the thickness of the 20 mm grain bed as the characteristic length, rather than the grain size, and the moisture ratio was normalized to the endpoint. Therefore, the numerical values and apparent activation energy characterize the configuration used in this experiment and should not be directly equated with the intrinsic mass transfer properties of the grains. The diffusion coefficient of rice grains increases with rising temperature [43], which is consistent with the results of this study. Other studies have also shown that the drying schedule can influence the estimated diffusion coefficient [44].

### 2.5. Fitting Performance of the Thin-Layer Drying Models

Each of the six thin-layer drying models was fitted separately to the 30 treatment datasets. Figure 6 and Table 3 compare their goodness of fit. Table 3 summarizes the mean *R*^2^ and *RMSE* values across the 30 treatment-specific fits for each model, while Figure 6 shows the *R*^2^ value for each model–treatment combination. The Midilli model performed best, with a mean *R*^2^ of 0.9988 and a mean *RMSE* of 0.0082. The Logarithmic model ranked second, with a mean *R*^2^ of 0.9861 and a mean *RMSE* of 0.0348. The Page, Henderson–Pabis, and Newton models also described the drying data reasonably well, with mean *R*^2^ values of at least 0.9803. The Wang–Singh model gave lower fitting accuracy under the normalization procedure used here. After accounting for the number of parameters, the Midilli model achieved the lowest *AIC* in 27 of the 30 treatments, further highlighting the model’s superior overall fitting performance; the Wang–Singh model achieved the lowest *AIC* in 3 of the treatments at 50 °C and 3 m/s.

The combination of exponential and linear terms in the Midilli model allows it to represent both the rapid initial decline and the slower subsequent change. Its superior in-sample performance makes it a useful candidate for detailed description of the present drying curves. The Page model offers a simpler alternative with fewer parameters and satisfactory fitting performance, which may favor practical implementation. The Logarithmic, Henderson–Pabis, and Newton models provide additional comparators, whereas the Wang–Singh model was less suitable for these data. Independent predictive performance cannot be established from these fits alone.

### 2.6. Main Effects of Drying Conditions on Quality Indicators

Table 4 presents the results of the main effect analysis for quality indicators under different drying conditions. The main effect of temperature on dry basis protein content reached a significant level (*p* = 0.011); the main effect of air velocity on dry basis amylose content was significant (*p* = 0.041), but this result did not remain significant in the HC3 sensitivity analysis. All other main effects did not reach a significant level.

For POD activity, the *p*-values for temperature, relative humidity, and air velocity were 0.230, 0.393, and 0.579, respectively; the corresponding values for SOD were 0.622, 0.523, and 0.799. None was below 0.05. Previous studies have also shown that the responses of drying performance and quality attributes are not necessarily consistent [45,46]. This further indicates that, within the range of drying conditions tested in this study, antioxidant enzyme activities are more likely to exhibit fluctuating responses under the combined effects of multiple factors, rather than a stable linear increase or decrease with any single factor.

To further examine the joint effects of factors beyond the main-effects model, a model including all two-factor interaction terms was additionally fitted. None of the joint tests of the two-factor interaction terms for protein, amylose, POD, or SOD were statistically significant. Residual diagnostics indicated that the residuals of the SOD model deviated from normality. In the HC3 sensitivity analysis, the temperature main effect on protein remained significant, whereas the *p*-value for the air velocity effect on amylose increased from 0.041 to 0.076, indicating that this effect was sensitive to the covariance estimator used.

### 2.7. Protein and Amylose Contents and Relative Retention

To further evaluate changes in nutritional composition, protein and amylose contents on a dry basis and their relative retentions were examined. Retention was calculated by assuming that dry matter mass remained unchanged within each treatment and that the composition of the undried reference represented the initial composition of all treatments. Values above 100% do not establish net formation of a constituent. The reference protein content was 8.03 g/100 g. Across the 30 treatments, dry basis protein content ranged from 7.83 to 8.26 g/100 g, with an overall mean of 7.999 g/100 g. Reference amylose content was 13.80%, whereas treatment values ranged from 10.50% to 16.90%, with a mean of 13.69%.

Dry basis protein and amylose contents and their relative retention at each temperature are summarized in Table 5 and Figure 7. Open blue circles and brown-orange triangles represent treatments at 3 and 4 m/s, respectively. Each temperature includes six combinations of three relative humidities and two air velocities; black diamonds and error bars indicate the mean ± sample standard deviation across these six combinations, and gray dashed lines indicate the undried reference values. Mean protein contents at 30, 35, 40, 45, and 50 °C were 8.013, 8.055, 7.965, 8.063, and 7.900 g/100 g, respectively. The lowest mean occurred at 50 °C, corresponding to a mean relative retention of 98.38%, whereas the highest mean occurred at 45 °C, with 100.42% relative retention. After Holm correction, pairwise comparisons showed that, under the conventional estimate, the protein contents at 35 °C and 45 °C were both higher than that at 50 °C; after applying the HC3 estimate, the difference between 45 °C and 50 °C remained significant (corrected *p* = 0.010). The lowest mean in the 50 °C group does not mean that it differs significantly from all other temperature groups, and the group means did not show a monotonic trend with increasing temperature. Although protein contents remained close to the reference level, the significant overall temperature effect indicated variation among temperature groups. Mean amylose content was highest at 35 °C (14.98%) and lowest at 50 °C (13.18%), corresponding to relative retentions of 108.57% and 95.53%, respectively. The relative variation in amylose content among treatment combinations was greater than that in protein. At 50 °C, both amylose and protein contents were relatively low and showed marked differences from the other temperature groups, providing a useful reference for the setting of drying process parameters.

Given the significant main effect of air velocity on amylose content, the results were also summarized by airflow condition. At 3 and 4 m/s, mean amylose contents were 14.31 ± 0.98% and 13.08 ± 2.04%, and mean relative retentions were 103.67% and 94.78%, respectively. The mean at 4 m/s was 1.23 percentage points lower than that at 3 m/s, equivalent to a relative decrease of 8.57%. Within the scope of this experiment and the model assumptions, increasing air velocity was associated with a lower measured amylose content [47], whereas the mechanisms linking air velocity and quality may differ across drying processes [48].

### 2.8. Changes in POD and SOD Activities

Building on the results of the multi-factorial main-effect analysis presented in Section 2.6, this section further examines the changes in peroxidase (POD) and superoxide dismutase (SOD) activities at different drying temperatures. As shown in Table 6, the POD and SOD activities of the undried paddy rice were 0.258 mU/g and 45.554 U/g, respectively. Within the temperature range of 30–50 °C, POD activity exhibited a non-monotonic pattern, with the highest mean value observed at 40 °C (0.266 ± 0.020 mU/g), corresponding to 103.23% of the activity in the undried paddy rice. At 45 and 50 °C, POD activity was maintained at 99.10% and 98.90% of the initial activity, respectively. In contrast, the mean SOD activities at all drying temperatures were lower than those of the undried paddy rice. Specifically, SOD activity at 50 °C was 43.132 ± 3.190 U/g, corresponding to a relative activity of 94.68%, whereas that at 40 °C was 42.339 ± 2.738 U/g, corresponding to 92.94% of the initial activity. These mean values reflect descriptive differences among the temperature groups; however, no statistically significant enzyme activation or inactivation was observed. Previous studies have demonstrated that drying temperature and initial moisture content jointly influence rice seed vigor, potentially involving reactive oxygen species (ROS) metabolism and antioxidant enzyme systems. Nevertheless, the underlying mechanisms require further investigation and validation [49,50].

Figure 8 provides an intuitive display of the changes in relative activities of POD and SOD at different temperatures. The dashed line indicates the 100% reference level, and the error bars represent the variation among the six humidity × air velocity combinations at each temperature. The dispersion of SOD at 35 °C was particularly large, corresponding to a sample standard deviation of 12.551 U/g in Table 6 and indicating the heterogeneity among the six humidity × air velocity combinations at this temperature.

### 2.9. Correlations Among Quality Indicators

To further clarify the correlation characteristics among quality indicators, pairwise Pearson correlation analyses were performed on the dry basis contents of protein and amylose, as well as the activities of POD and SOD, for the 30 treatments. Table 7 lists all six pairs of Pearson correlations calculated based on the means of the 30 treatments. The Holm correction was applied to the correlation tests, and significance was judged by the corrected *p*-values.

Neither protein nor amylose content was significantly correlated with POD activity, and the correlation between POD and SOD was also nonsignificant (r = 0.116, *p* = 0.540). In contrast, protein content was negatively correlated with SOD activity (*p* < 0.001), as was amylose content (*p* = 0.041). Among the two negative correlations, the absolute value of the correlation coefficient between protein and SOD was relatively large. After Holm correction, only the negative correlation between protein and SOD remained significant (corrected *p* = 0.003), whereas the association between amylose and SOD did not reach a significant level (corrected *p* = 0.207). Protein and amylose contents were positively correlated, but this association did not reach statistical significance. The correlation analysis described in this study cannot serve as evidence that SOD causes nutrient loss or that protein content regulates SOD, but it can be interpreted as an exploratory association between them [51,52]. The mechanisms underlying these associations require further investigation using catalytic activity assays and measurements of oxidative damage [53].

## 3. Discussion

Based on thin-layer drying experiments conducted on paddy rice under combined stresses, this study used characteristic parameters, including *M*_r_, MR^*^, D_eff_^*^, and water-loss rate to examine moisture migration in relation to changes in temperature, relative humidity, and air velocity. Six thin-layer drying models (Newton, Page, Henderson–Pabis, Logarithmic, Wang–Singh, and Midilli) were evaluated in terms of their goodness of fit and potential value for practical drying operations. Main-effect analyses of the drying factors further elucidated changes in protein content, amylose content, and the activities of the antioxidant enzymes peroxidase (POD) and superoxide dismutase (SOD). Pairwise correlations among these four quality indicators were also examined. These findings provide a theoretical basis and experimental evidence for optimizing paddy rice drying under combined stresses, controlling moisture migration, and preserving quality and antioxidant enzyme activity.

As the temperature increased from 30 to 50 °C, the mean drying time decreased, while the mean water-loss rate and *D*_eff_^*^ increased significantly, indicating that higher temperatures improved dehydration efficiency under the thin-layer conditions investigated. However, the physical interpretation of these findings must take the experimental configuration into account. D_eff_^*^ was calculated using a characteristic length of 0.01 m, corresponding to half the bed thickness. It is therefore an apparent parameter for the bed configuration used in this study and can serve only as a reference for characterizing the intrinsic moisture diffusivity within individual paddy rice kernels [54,55]. Within the ranges investigated, changes in relative humidity and air velocity did not produce clear patterns in moisture migration during drying. Further experiments will consider broader ranges of these stress factors [56].

Among the six thin-layer drying models, the Midilli model achieved the highest mean *R*^2^ and the lowest mean *RMSE* across the 30 datasets. Comparisons of the mean evaluation metrics placed the Logarithmic model second and the Page model third. These results indicate that the Midilli model provided the most accurate fit to the apparent moisture ratio curves within the experimental range, highlighting its value for practical drying applications. The Midilli model has a relatively large number of adjustable parameters, whereas drying at higher temperatures involved shorter drying times and fewer weighing time points. Model flexibility may therefore have contributed to its high goodness of fit. The Page model also merits consideration for practical applications because of its simple mathematical form and good fitting performance [57,58,59]. Nevertheless, this study characterizes only the descriptive ability of these models; their predictive performance on independent data will be evaluated in future work [60,61].

Protein content remained broadly comparable to that of the undried grain, although the mean was lowest at 50 °C. The overall main effect of temperature was significant, but this does not establish a significant difference between every pair of temperature treatments. The extent of protein denaturation also remains unresolved, as neither protein structure nor digestibility was examined; these measurements will be included in future work [62]. Amylose content fluctuated considerably among different operating conditions. Although the main effect of air velocity reached a significant level, it did not remain significant after the HC3 sensitivity analysis. The effects of process sequence on drying rate, amylose content, and processing and eating quality were not entirely consistent, suggesting that the evaluation of drying processes needs to consider multiple indicators simultaneously [63,64,65]. Neither POD nor SOD activity followed a consistent monotonic pattern as temperature increased. No significant main effect of any of the three drying factors was detected for either enzyme. This finding is specific to the sample size and additive model used here and should not be taken as evidence that enzyme activity is unaffected by drying conditions [66,67,68]. Measurements of reactive oxygen species, membrane damage, and related metabolic indicators were not available in this study. Including them in future experiments could help clarify the antioxidant regulatory mechanisms involved in different drying processes. In the pairwise Pearson analysis of the four quality indicators, protein content was significantly negatively correlated with SOD activity (Holm-corrected *p* = 0.003), whereas the correlation between amylose and SOD activity did not reach a significant level after Holm correction. These results provide a basis for examining the different responses of protein content and antioxidant enzyme activities during drying and for informing subsequent process control. They also suggest a possible association between SOD activity and the nutritional quality indicators of paddy rice, which warrants further evaluation in studies with larger sample sizes.

Overall, this study has certain limitations in its experimental design, which also highlight directions for further research. From an experimental perspective, future studies could incorporate independent replicate drying experiments, increase the number of material batches, and shorten sampling intervals, particularly under higher-temperature conditions, to more comprehensively characterize the dynamic changes in moisture content and quality indicators during drying. At the modeling level, conducting grain-scale measurements and employing models that incorporate appropriate geometrical representations and boundary conditions would help improve the interpretation of moisture migration behavior. Furthermore, before conducting multi-response optimization, independent predictive validation, direct measurements of enzyme activities, assessments of oxidative damage, and evaluations of milling or sensory quality endpoints should be considered to enhance the robustness and practical applicability of the conclusions. Notably, based on the findings of the present study, drying temperatures of 35–40 °C may serve as a candidate range for further comparative investigations aimed at identifying optimal conditions that balance multiple objectives or developing validated intelligent drying-control strategies. This direction appears feasible and warrants further exploration.

## 4. Materials and Methods

### 4.1. Overview of the Study Area

The paddy rice samples used in this study were of the Koshihikari cultivar and were grown in Yingkou City, Liaoning Province, China. Yingkou City is located in the southern part of Northeast China, in the lower reaches of the Liaohe River Plain, at approximately 121°56′–123°02′ E and 39°55′–40°56′ N. It has a warm temperate continental monsoon climate, with four distinct seasons and rainfall concentrated during the warm season. The annual average temperature is about 9–10 °C, the annual precipitation is about 600–700 mm, and the frost-free period is about 170–180 days; the light and heat conditions are relatively suitable for rice growth. The soils in this region are mainly paddy soils and meadow soils, with relatively deep soil layers, relatively high organic matter content, and sufficient irrigation water sources, making it one of the important high-quality rice production areas in Northeast China. The paddy rice harvest period is usually concentrated from late September to mid-October each year, and after harvest, drying treatment is often required to reduce the moisture content and ensure safe storage. The Koshihikari cultivar selected in this study is widely cultivated in this region and has strong regional representativeness [69]. Studying moisture migration and the associated quality changes during the drying of this paddy rice under multi-factor stress can help guide the optimization of drying practices in the region.

### 4.2. Experimental Materials

The samples used for testing were freshly harvested Koshihikari paddy rice from the Yingkou region in October 2025. Fully developed grains of similar maturity, without visible mechanical damage, mold, or insect infestation, were selected. The initial moisture content was 17.4% on a wet basis. The samples were stored at a constant temperature of 4 °C and returned to room temperature before the start of the experiment. For each treatment, a 700 g paddy rice sample was spread evenly on a drying tray to form a 20 mm thick bed. Drying experiments were conducted using a JK-KB1700 thin-layer hot-air drying simulator (Jilin University, Changchun City, China).

### 4.3. Experimental Design

A full factorial design was used, with drying temperature, relative humidity, and air velocity as the experimental factors. As summarized in Table 8, five drying temperatures (30, 35, 40, 45, and 50 °C), three relative humidity levels (20%, 25%, and 30%), and two air velocities (3 and 4 m/s), regulated by adjusting the equipment operating frequency, were combined to yield 30 planned treatment combinations. The material thickness is an important characteristic length in this study and will be used subsequently to estimate the apparent effective moisture diffusivity (${D_{\mathrm{eff}}}^{*}$).

A total of 33 drying runs were performed. Three runs were deemed invalid because of weighing or time-recording errors and were excluded. Each affected treatment was subsequently repeated in full under the originally specified conditions. The final dataset contained one valid drying run for each of the 30 combinations; independent replicate drying runs were not retained within the same treatment, and technical replicates were not treated as independent drying replicates. The undried grain sample served as the reference for normalizing the quality indicators; it was excluded from the main-effect and correlation analyses and was not counted among the experimental treatments.

Before each experiment, the drying apparatus was set to the specified temperature, relative humidity, and air velocity and allowed to reach stable operating conditions. The sample tray was then placed in the chamber. The target endpoint was a wet-basis moisture content of 14.0%, with some deviation permitted. Sample mass was recorded at 10 min intervals until the prescribed endpoint was reached. After drying, samples were placed in labeled, sealed bags and temporarily stored at 4 °C pending analysis. A 50 g subsample was retained for POD and SOD assays, and 300 g was retained for starch, protein, and fat analyses. The protein content, amylose content, POD activity, and SOD activity of rice grains were determined in technical triplicate for each sample, and the mean of the three technical replicates was taken as the final observed value for each treatment. Technical replicates were not counted as independent experimental replicates or biological replicates, as the design included only one independent dried sample per treatment combination. Figure 9 presents the experimental samples, with subpanels a, b, and c showing the freshly harvested wet rice grain samples, the dried rice grain samples, and the brown rice samples obtained after dehulling the dried rice grains, respectively.

### 4.4. Measurements and Calculations

#### 4.4.1. Moisture Content

Moisture content was determined according to the Chinese national standard GB/T 5497-1985 [70], Inspection of grain and oilseeds—Methods for determination of moisture content.

#### 4.4.2. Moisture-Migration Indicators

The mass ratio (*Mr*), apparent moisture ratio (MR^*^), and mean water-loss rate (*V*) were calculated to characterize mass reduction and moisture migration during drying:

(1) $$\mathrm{Mr}=\frac{M_{t}}{M_{0}}$$

(2) $${\mathrm{MR}}^{*}=\frac{M_{t}-M_{e}}{M_{0}-M_{e}}$$

*M_t_* is the sample mass at drying time t (g);

*M*_0_ is the initial sample mass (g);

*M_e_* is the sample mass at the final weighing, used as the endpoint reference mass (g).

(3) $$V=\frac{M_{0}-M_{f}}{t^{d}}$$

*M_f_* is the sample mass at the end of drying (g);

*t^d^* is the total drying time (min).

#### 4.4.3. Quality Measurements

Protein content was determined using the Kjeldahl method (Method I) specified in GB 5009.5-2016, National Food Safety Standard—Determination of Protein in Foods [71]. Amylose content was determined using the defatting procedure specified in GB/T 15683-2025, Inspection of Grain and Oils—Determination of Amylose Content in Rice [72]. Relative protein and amylose retention were expressed as percentages of the corresponding dry basis contents in the undried reference sample. Relative POD and SOD activities were calculated using the measured activity in the reference sample as 100%. The measured protein content was reported on a wet basis and converted to a dry basis using Equation (4):

(4) $$\mathrm{Cdb}=\frac{\mathrm{Cwb}}{1-W_{f}/100}$$

*Cdb* is the dry basis protein content (g/100 g);

*Cwb* is the measured wet-basis protein content (g/100 g);

*W_f_* is the final wet-basis moisture content (%).

SOD and POD activities were measured using enzyme assay kits produced by Shanghai Kexing Trading Co., Ltd. (Shanghai, China), and the microplate reader used was a Labsystems Multiskan MS 352 (Labsystems, Finland) [73]. According to the kit instructions, target-specific capture antibodies and HRP-labeled detection antibodies were used, followed by TMB color development. The POD standard levels were 1.5, 3, 6, 12, and 24 mU/L, and the SOD standard levels were 250, 500, 1000, 2000, and 4000 U/L. These units are those of the calibrators provided in the kits, and this method does not directly measure the catalytic reaction rate of the sample enzymes. For the standard wells, 50 μL of standard was added to each well; for the sample wells, 10 μL of sample extract and 40 μL of diluent were added. The plates were incubated at 37 °C for 30 min and washed five times. Subsequently, 50 μL of HRP-labeled reagent was added, followed by another incubation at 37 °C for 30 min and five washes. Next, color developers A and B (50 μL each) were added, and the plates were incubated at 37 °C in the dark for 10 min to allow color development. After 50 μL of stop solution was added, absorbance was measured at 450 nm within 15 min using a Labsystems Multiskan MS 352 microplate reader.

### 4.5. Construction of a 3D Interpolation Surface for Average Moisture Loss Rate

To visualize how mean water-loss rate varied with temperature, relative humidity, and air velocity, three-dimensional interpolation surfaces were constructed from the measured values for each experimental condition. Bivariate spline interpolation is a numerical method based on piecewise polynomials that constructs a continuous surface with a specified degree of smoothness from a finite set of discrete observations. It has been widely used in the numerical analysis and visualization of multidimensional data [74].

Because only two discrete air velocity levels were investigated, separate interpolation surfaces were constructed for 3 and 4 m/s, each using 15 measured data points. The RectBivariateSpline function in the SciPy library was used to construct the surfaces, with temperature and relative humidity as the independent variables and mean water-loss rate as the response [75]. A bivariate tensor-product B-spline formulation was adopted, with cubic splines along the temperature axis (*kx* = 3), which comprised five experimental levels, and quadratic splines along the relative humidity axis (*ky* = 2), which comprised three experimental levels. These spline degrees were selected to accommodate the number of data points along each axis. The smoothing parameter was set to s = 0 so that the resulting surfaces passed through all measured data points. Interpolation was restricted to the experimental ranges of the factors, and no extrapolation was performed.

### 4.6. Estimation of Diffusion Parameters

Estimation of moisture diffusivity can support the selection of drying conditions and the evaluation of drying efficiency and energy use. Apparent effective moisture diffusivity (${D_{\mathrm{eff}}}^{*}$) was estimated from the linear relationship between lnMR^*^ and drying time, t, using a slab approximation derived from Fick’s second law [76]. The goodness of the linear fit was evaluated using *R*^2^:

(5) $$\ln{\mathrm{MR}}^{*}=\ln\frac{8}{\pi^{2}}-\frac{{D_{\mathrm{eff}}}^{*}\pi^{2}t}{4L^{2}}$$

*L* is half the grain-bed thickness; in this study, *L* = 0.01 m;

*t* is drying time (s).

If *k* denotes the slope of this linear relationship, apparent effective moisture diffusivity is calculated as follows:

(6) $${D_{\mathrm{eff}}}^{*}=-\frac{4L^{2}}{\pi^{2}}k$$

The relationship between ${D_{\mathrm{eff}}}^{*}$ and temperature was fitted using the Arrhenius equation to estimate the apparent activation energy (*E_a_*) and assess the temperature sensitivity of moisture migration:

(7) $${D_{\mathrm{eff}}}^{*}=D_{0}\exp(-\frac{E_{a}}{RT})$$

*D*_0_ is the pre-exponential factor (m^2^/s), representing the extrapolated diffusivity as absolute temperature approaches infinity;

R is the universal gas constant, R = 8.314 × 10^−3^ kJ/(mol·K);

*T* is absolute temperature (K), *T* = *t* + 273.15.

### 4.7. Drying-Model Fitting

The Newton, Page, Henderson–Pabis, Logarithmic, Wang–Singh, and Midilli models were fitted to characterize thin-layer drying kinetics. Their equations are listed in Table 9.

Model performance was evaluated using the coefficient of determination (*R*^2^) and root mean square error (*RMSE*). In addition, the Akaike information criterion (*AIC*) was used as a supplementary metric to compare the drying models while accounting for model complexity and the risk of overfitting associated with additional parameters. A lower *AIC* indicates a more favorable balance between goodness of fit and model complexity. *AIC* was calculated as follows:

*AIC* = 2*n* − 2ln(L) (14)

*n* is the number of estimated model parameters;

*L* is the maximized value of the likelihood function.

### 4.8. Statistical Analysis

Quality measurements are summarized as means ± standard deviations (SDs). The mean of three technical measurements for each treatment sample was used in the statistical analyses; technical replicates were not treated as independent drying replicates. Temperature-specific means and SDs were calculated from the treatment means of the six relative humidity ×air velocity combinations at each temperature, thereby reflecting variation among different treatment combinations. The undried grain served solely as the normalization reference and was excluded from the main-effect and correlation analyses.

Temperature, relative humidity, and air velocity were treated as categorical fixed factors. Additive general linear models were used to evaluate their main effects on protein content, amylose content, POD, and SOD. Interactions were additionally assessed using partial F-tests comparing nested models with and without two-factor interaction terms. For ${D_{\mathrm{eff}}}^{*}$, ln(${D_{\mathrm{eff}}}^{*}$) was modeled as a function of the three main effects and all two-factor interactions. Because only one valid drying run was retained for each treatment combination, the interaction tests relied on the assumption that the unmodeled three-factor interaction was negligible, and their results were interpreted as exploratory evidence.

Residual normality was assessed using the Shapiro–Wilk test, and homogeneity of residual variances across the levels of each factor was examined using the median-centered Levene test. Following the significant temperature main effect on protein content, pairwise comparisons among temperature levels were performed using the additive model, with Holm adjustment for multiple testing. No additional post hoc comparisons were performed for air velocity because it comprised only two levels. Sensitivity analyses using the HC3 heteroscedasticity-robust covariance estimator were also conducted for the main effects on quality indicators and the pairwise temperature comparisons for protein content.

Pearson correlation analysis [77,78] was used to evaluate linear associations among protein content, amylose content, POD, and SOD. Holm adjustment was applied to all six pairwise correlation tests. The correlations were interpreted as exploratory associations, without causal inference. Statistical significance was defined as *p* < 0.05; adjusted *p*-values were used when multiple comparisons were performed.

## 5. Conclusions

This study investigated Koshihikari paddy rice through a series of thin-layer hot-air drying experiments under multiple stress conditions. The effects of drying temperature, relative humidity, and air velocity on moisture migration behavior were systematically examined, and changes in starch and protein contents and antioxidant enzyme activities during drying were explored. In addition, the fitting performance and applicability of six thin-layer drying models were comparatively evaluated.

The results demonstrated that increasing the drying temperature improved the thin-layer drying efficiency of paddy rice within the investigated range. As the temperature increased from 30 to 50 °C, the mean drying time decreased from 125.00 to 48.33 min, while the mean water-loss rate increased from 0.204 to 0.662 g/min. The interpolated water-loss-rate surfaces exhibited an upward trend with increasing temperature, accompanied by relatively minor fluctuations associated with changes in relative humidity and air velocity. Meanwhile, the mean bed-scale apparent effective moisture diffusivity Deff* increased from 1.572 × 10^−8^ to 3.843 × 10^−8^ m^2^/s.

Regarding model fitting, the Midilli model exhibited the best in-sample fitting performance across the 30 experimental datasets, with a mean coefficient of determination (*R*^2^) of 0.9988 and a mean root mean square error (*RMSE*) of 0.0082. It also generally yielded the lowest Akaike information criterion (*AIC*) values, indicating its superior applicability for detailed fitting of drying kinetics. The Logarithmic model ranked second, with a mean *R*^2^ of 0.9861 and a mean *RMSE* of 0.0348. The Page model also achieved a relatively high *R*^2^, while its simpler structure further enhanced its potential for practical drying-process applications.

Regarding quality responses, temperature had a significant main effect on protein content (*p* = 0.011), with the lowest mean value observed at 50 °C (7.900 g/100 g). The main effect of air velocity on amylose content reached a significant level (*p* = 0.041), but this result did not remain significant in the HC3 sensitivity analysis. In contrast, none of the three drying factors—temperature, relative humidity, or air velocity—exhibited a significant main effect on peroxidase (POD) or superoxide dismutase (SOD) activity. These findings suggest that protein and amylose contents were more sensitive to variations in drying conditions than antioxidant enzyme activities within the investigated experimental range. Correlation analysis showed that the Pearson correlation coefficients of protein and amylose with SOD were −0.595 (*p* < 0.001) and −0.375 (*p* = 0.041), respectively. After Holm correction, only the negative correlation between protein and SOD remained significant; none of the other five pairwise correlations was significant.

Overall, increasing temperature improved drying efficiency within the experimental range, although the quality indicators responded differently. Considering the observed changes in drying performance and quality, 35–40 °C may be considered a candidate temperature range for balancing dehydration efficiency with the retention of nutritional components and antioxidant enzyme activities, providing a reference for further process optimization. Its overall advantages require further experimental validation. These findings provide an empirical basis and theoretical support for the multi-objective optimization of paddy rice drying.

## Figures and Tables

**Figure 1:**
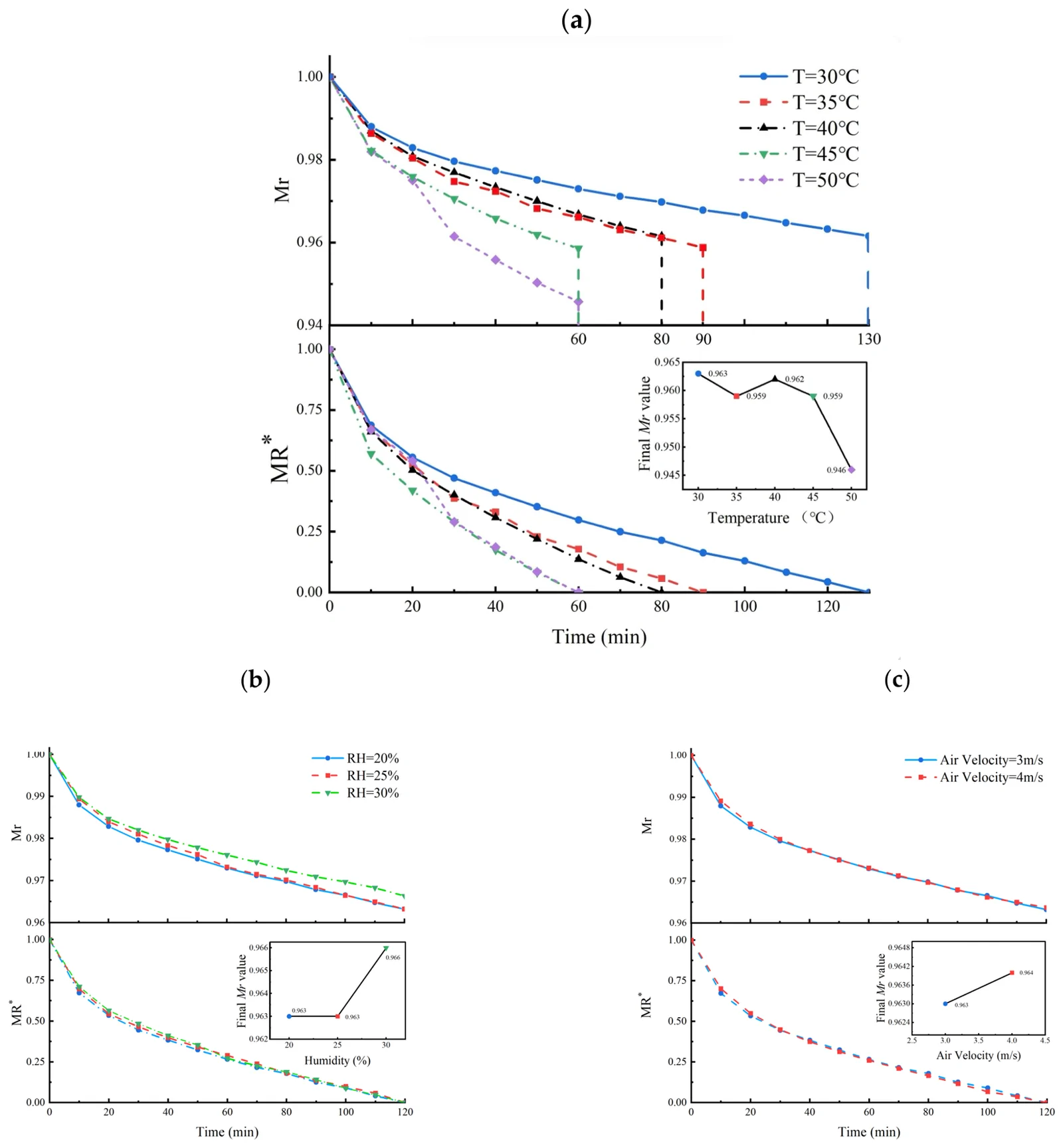
Comparison of mass ratio and apparent moisture ratio under different (**a**) temperatures, (**b**) relative humidities, and (**c**) air velocities. (**a**) Temperature: RH = 20%, air velocity = 3 m/s. (**b**) Relative humidity: temperature = 30 °C, air velocity = 3 m/s. (**c**) Air velocity: temperature = 30 °C, RH = 20%.

**Figure 2:**
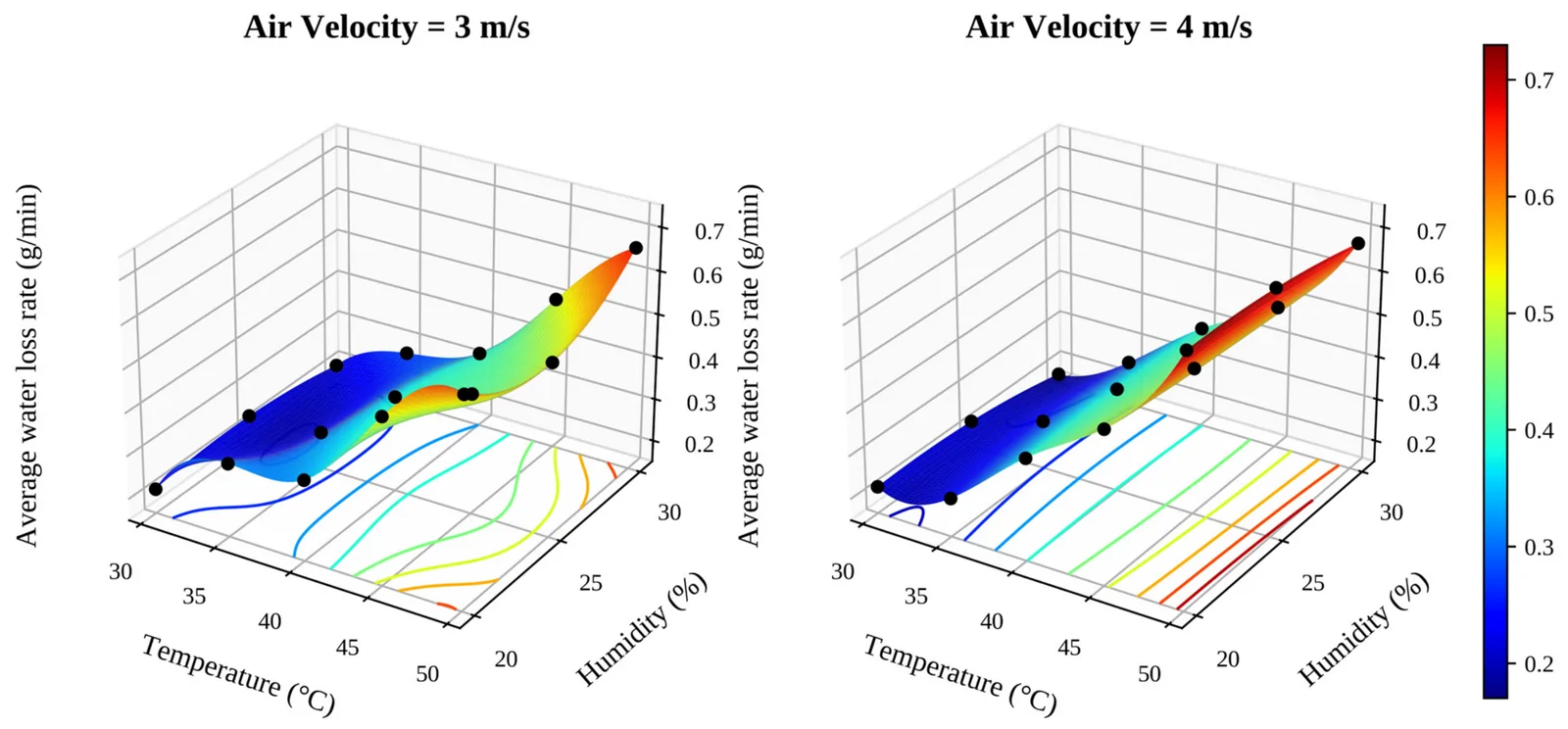
Three-dimensional interpolation surfaces of mean water-loss rate.

**Figure 3:**
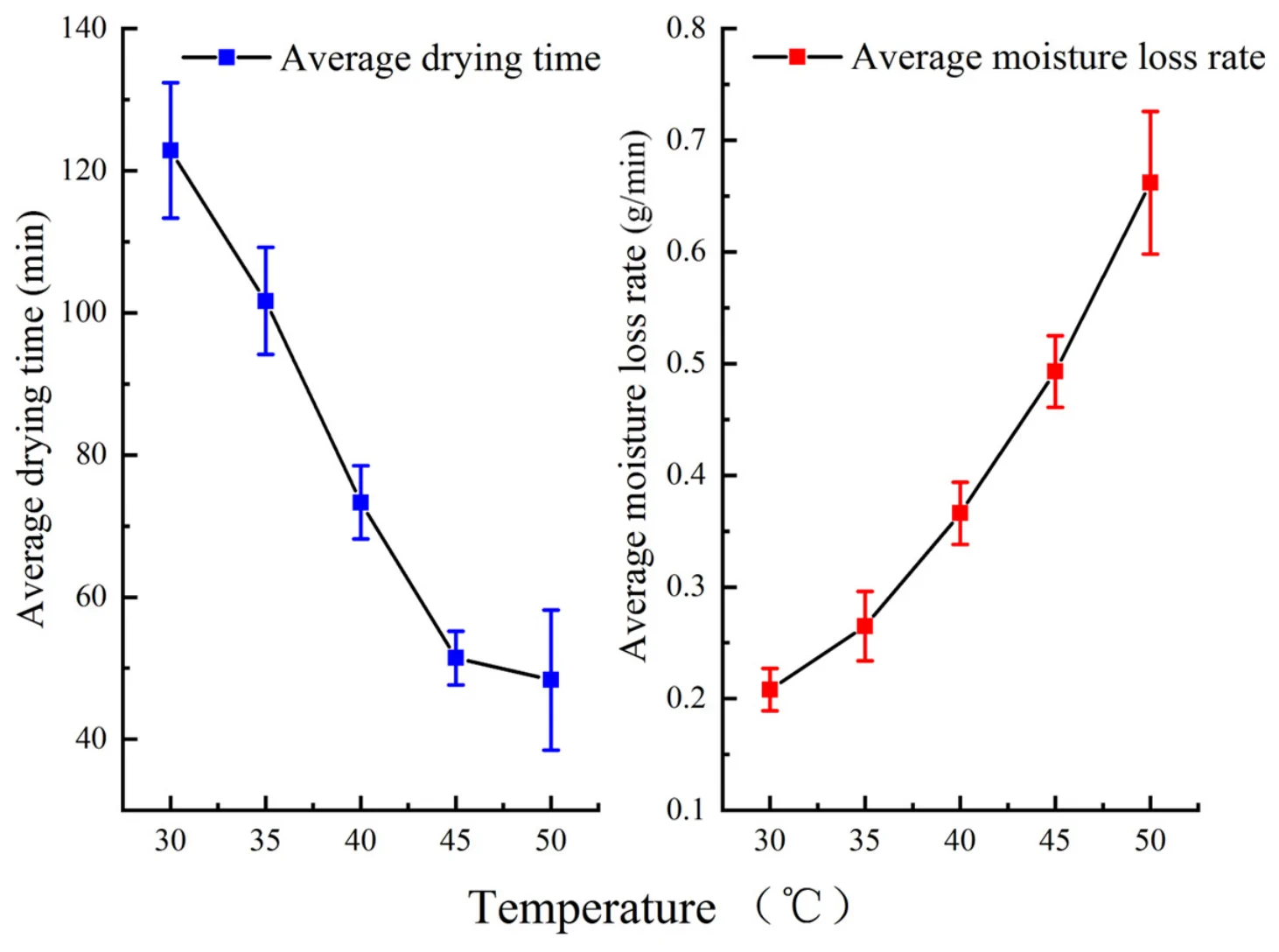
Mean drying time and mean water-loss rate at different drying temperatures.

**Figure 4:**
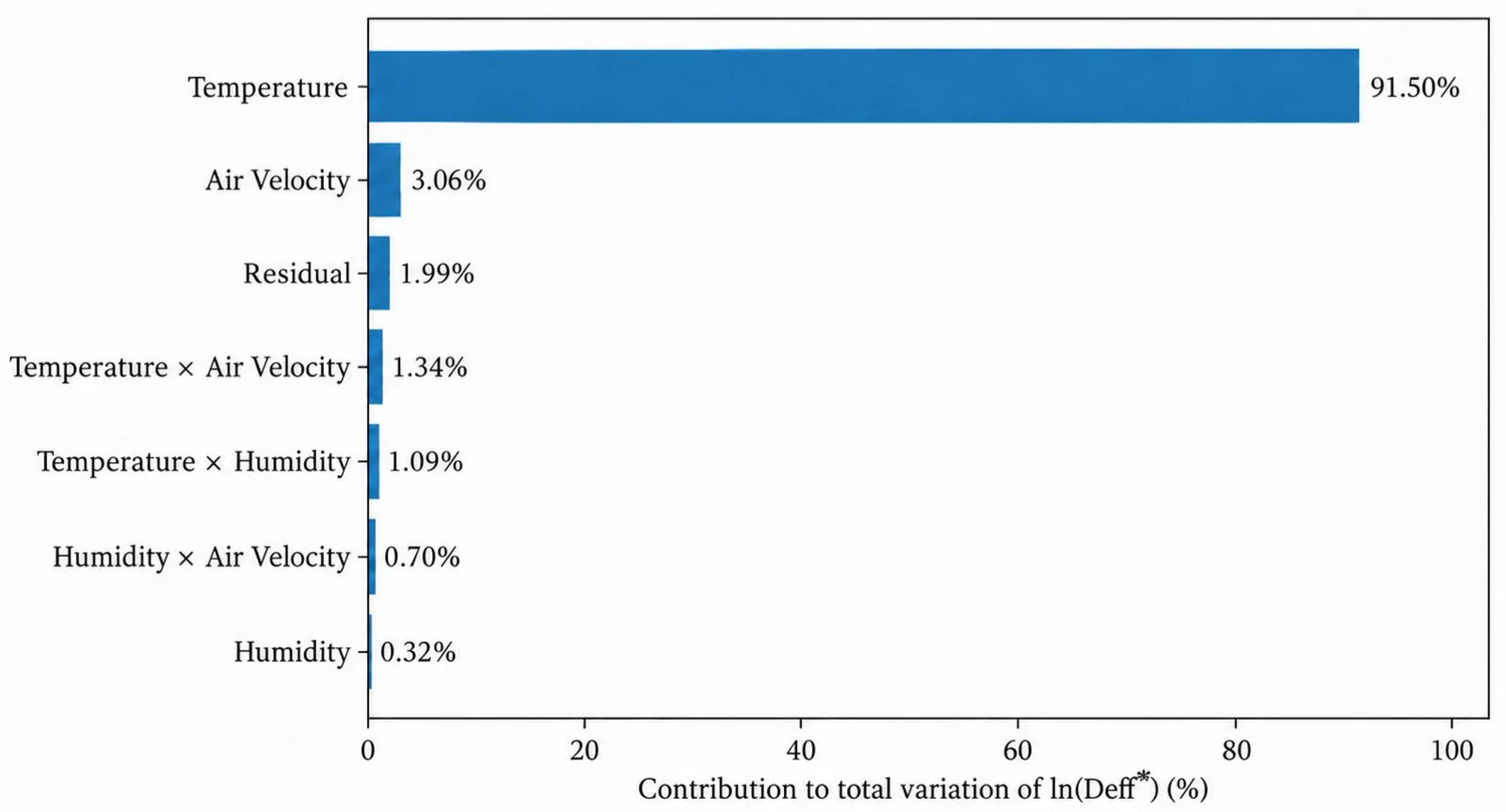
Contributions of drying factors and two-factor interactions to the corrected total variation in ln(D_eff_^*^).

**Figure 5:**
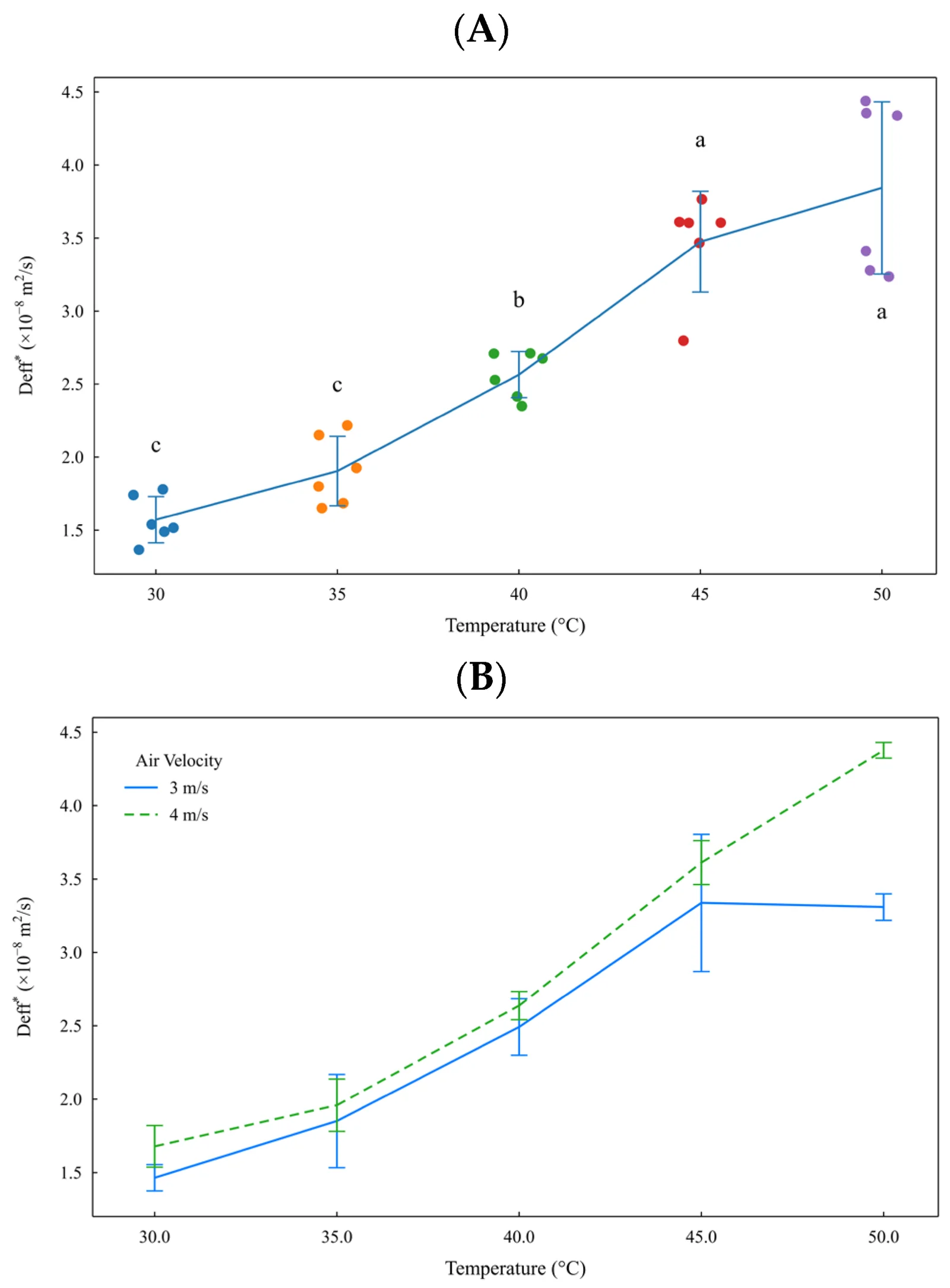
Variation in apparent effective moisture diffusivities. (**A**) Effect of temperature on ${D_{\mathrm{eff}}}^{*}$. (**B**) Interactive effect of temperature × air velocity on ${D_{\mathrm{eff}}}^{*}$.(Letters a, b, and c indicate whether the differences are significant; the same letter indicates no significant difference.).

**Figure 6:**
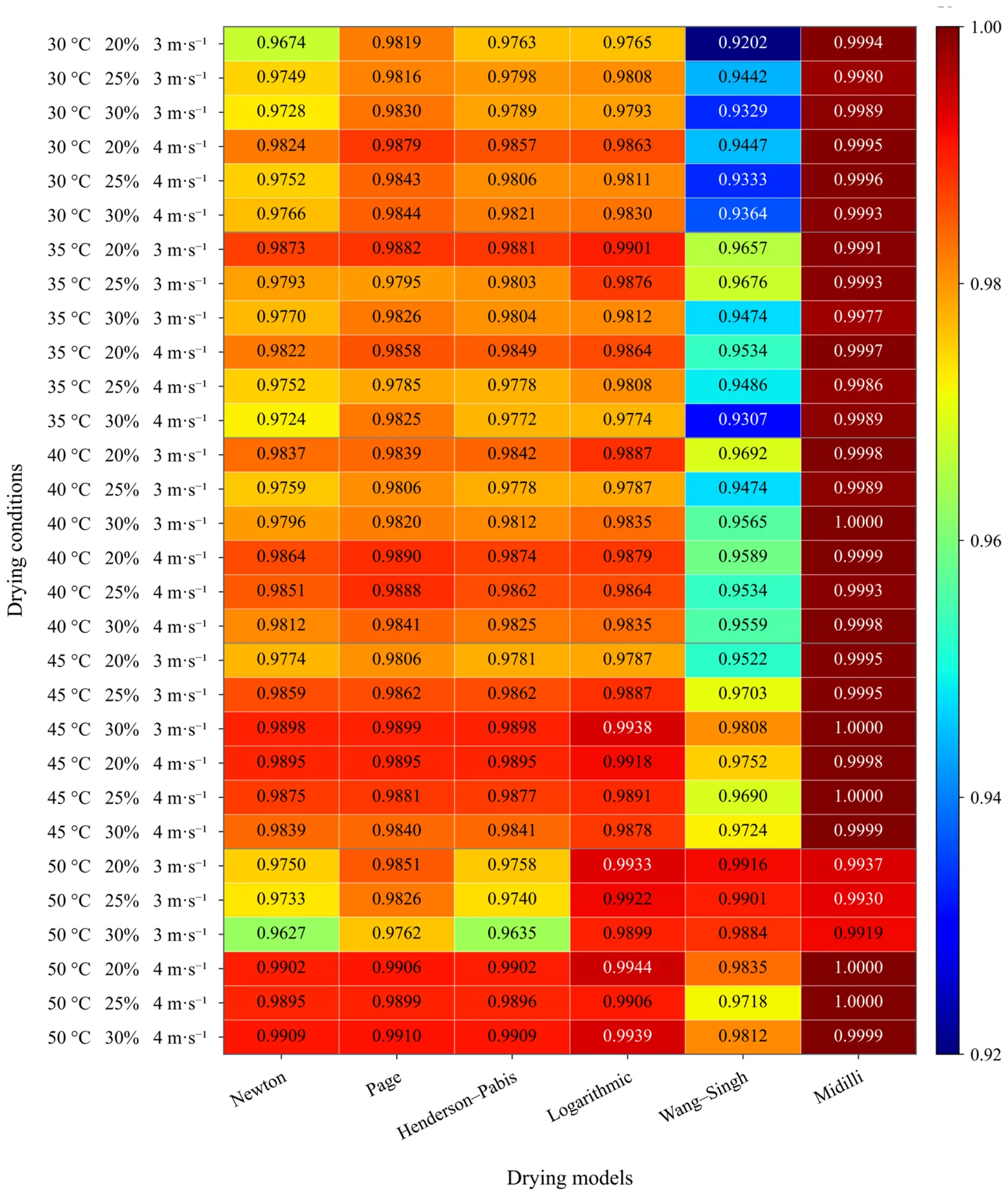
Comparison of the goodness of fit of six thin-layer drying models across the 30 treatments. (*R*^2^ values in the figure are rounded).

**Figure 7:**
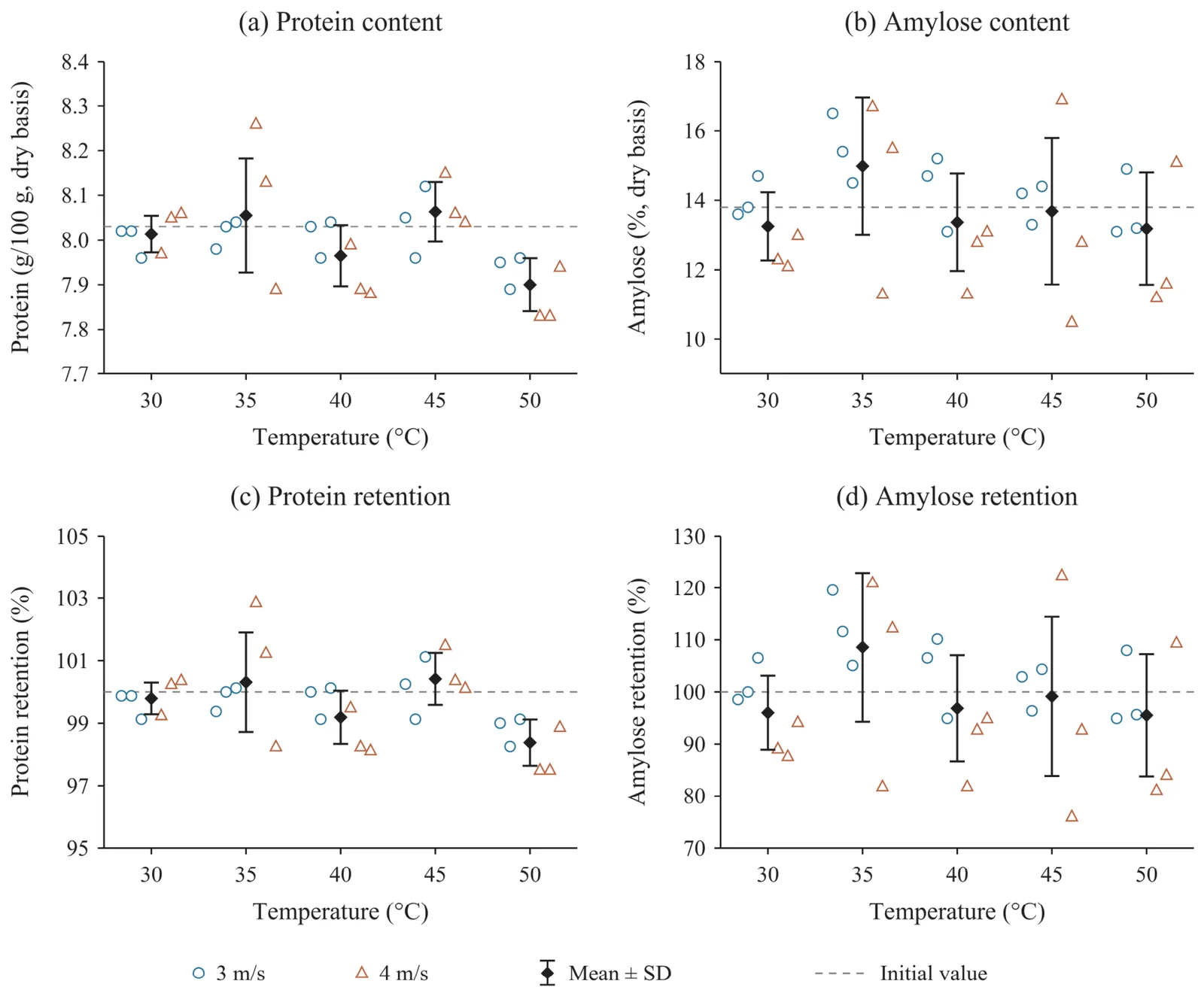
Visual analysis of protein and amylose contents and quality retention of paddy rice.

**Figure 8:**
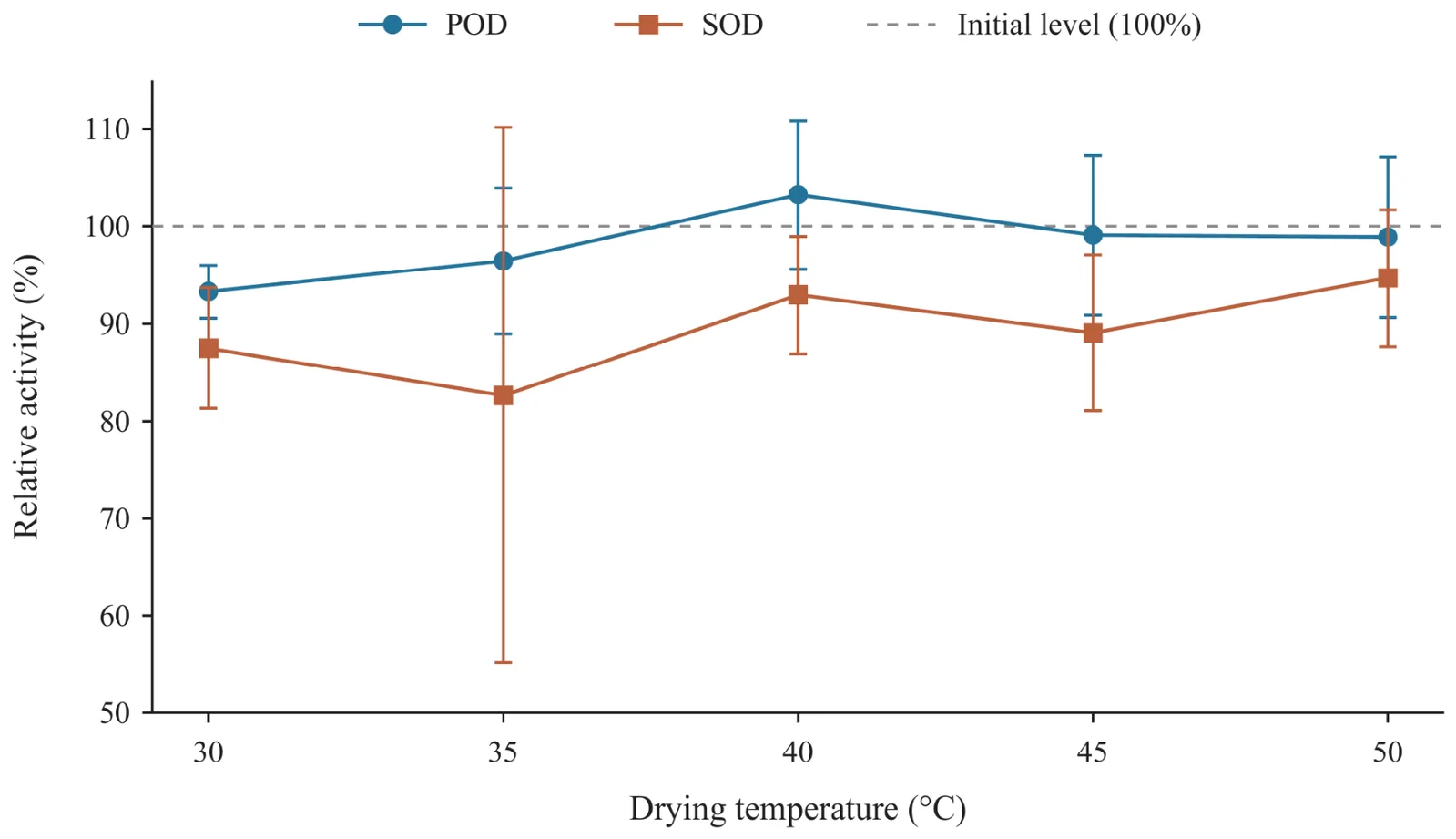
Relative POD and SOD activities at different drying temperatures.

**Figure 9:**
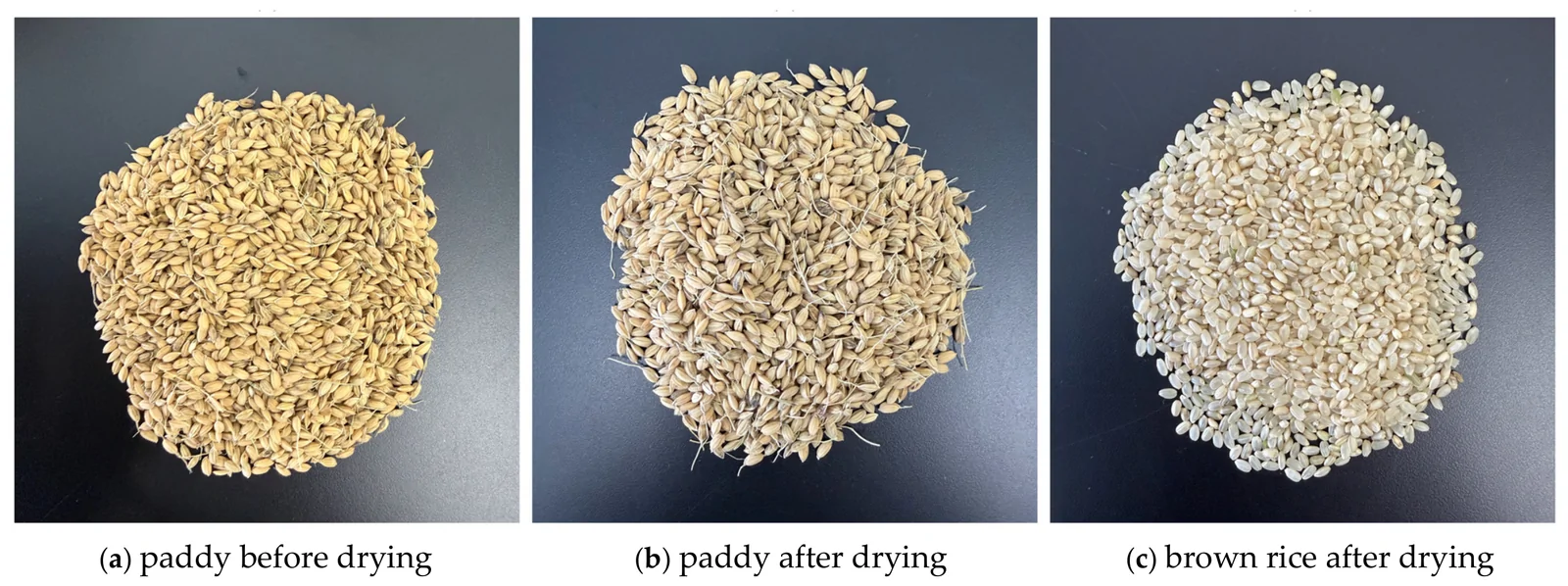
Photographs of the experimental materials.

**Table 1:** Drying characteristics of paddy rice at different temperatures.

Temperature (°C)	Mean Drying Time (min)	Water-Loss (g)	Mean Water-Loss Rate (g/min)
30	125.00 ± 8.37	25.36	0.204 ± 0.017
35	101.67 ± 7.53	26.71	0.265 ± 0.031
40	73.33 ± 5.16	26.73	0.366 ± 0.028
45	51.67 ± 4.08	25.22	0.490 ± 0.034
50	48.33 ± 9.83	31.57	0.662 ± 0.064

**Table 2:** ${D_{\mathrm{eff}}}^{*}$ and goodness of fit for the 30 drying treatments.

Temperature (°C)	Humidity (%)	Air Velocity (m/s)	D_eff_^*^ (m^2^/s)	*R* ^2^	Mean D_eff_^*^ (m^2^/s)	Mean *R*^2^
30	20	3	1.490 × 10^−8^	0.9507	1.572 × 10^−8^	0.9579
	25		1.366 × 10^−8^	0.9802		
	30		1.539 × 10^−8^	0.9701		
	20	4	1.779 × 10^−8^	0.9543		
	25		1.740 × 10^−8^	0.9434		
	30		1.516 × 10^−8^	0.9487		
35	20	3	2.217 × 10^−8^	0.9736	1.905 × 10^−8^	0.9552
	25		1.685 × 10^−8^	0.9501		
	30		1.651 ×10^−8^	0.9823		
	20	4	1.799 × 10^−8^	0.9699		
	25		2.151 × 10^−8^	0.9001		
	30		1.926 × 10^−8^	0.9549		
40	20	3	2.415 × 10^−8^	0.9591	2.565 × 10^−8^	0.9766
	25		2.711 × 10^−8^	0.9801		
	30		2.350 × 10^−8^	0.9685		
	20	4	2.675 × 10^−8^	0.9866		
	25		2.709 × 10^−8^	0.9816		
	30		2.528 × 10^−8^	0.9833		
45	20	3	3.610 × 10^−8^	0.9745	3.475 × 10^−8^	0.9861
	25		2.798 × 10^−8^	0.9914		
	30		3.604 × 10^−8^	0.9904		
	20	4	3.765 × 10^−8^	0.9902		
	25		3.605 ×10^−8^	0.9897		
	30		3.467 × 10^−8^	0.9806		
50	20	3	3.237 × 10^−8^	0.9707	3.843 × 10^−8^	0.9805
	25		3.279 × 10^−8^	0.9643		
	30		3.411 × 10^−8^	0.9636		
	20	4	4.356 × 10^−8^	0.9957		
	25		4.438 × 10^−8^	0.9919		
	30		4.339 × 10^−8^	0.9969		

**Table 3:** Mean goodness-of-fit statistics for the six thin-layer drying models.

Models	Mean *R*^2^	Mean *RMSE*
Midilli	0.9988	0.0082
Logarithmic	0.9861	0.0348
Page	0.9848	0.0372
Henderson–Pabis	0.9824	0.0398
Newton	0.9803	0.0417
Wang–Singh	0.9598	0.0581

**Table 4:** Main-effect *p*-values for temperature, relative humidity, and air velocity in the quality-indicator models.

Quality Indicator	Temperature	Relative Humidity	Air Velocity
Protein (dry basis)	0.011 */0.011 *	0.512/0.614	0.929/0.939
Amylose (dry basis)	0.268/0.408	0.334/0.438	0.041 */0.076
POD	0.230/0.126	0.393/0.375	0.579/0.634
SOD	0.622/0.638	0.523/0.655	0.799/0.827

* *p* < 0.05. Each cell presents the *p*-values corresponding to the conventional covariance estimate and the HC3 heteroskedasticity-robust covariance estimate, respectively.

**Table 5:** Dry basis protein and amylose contents and their relative retention at different drying temperatures.

Temperature (°C)	Protein, Dry Basis (g/100 g)	Protein Retention (%)	Amylose, Dry Basis (%)	Amylose Retention (%)
Undried reference	8.03	100.00	13.80	100.00
30	8.013 ± 0.041	99.79 ± 0.51	13.25 ± 0.98	96.01 ± 7.11
35	8.055 ± 0.128	100.31 ± 1.59	14.98 ± 1.97	108.57 ± 14.31
40	7.965 ± 0.068	99.19 ± 0.85	13.37 ± 1.41	96.86 ± 10.18
45	8.063 ± 0.067	100.42 ± 0.83	13.68 ± 2.11	99.15 ± 15.27
50	7.900 ± 0.059	98.38 ± 0.74	13.18 ± 1.62	95.53 ± 11.72

**Table 6:** POD and SOD activities and their relative activities at different drying temperatures.

Temperature (°C)	POD (mU/g)	Relative POD Activity (%)	SOD (U/g)	Relative SOD Activity (%)
Undried reference	0.258	100.00	45.554	100.00
30	0.241 ± 0.007	93.28	39.866 ± 2.809	87.51
35	0.249 ± 0.019	96.45	37.646 ± 12.551	82.64
40	0.266 ± 0.020	103.23	42.339 ± 2.738	92.94
45	0.256 ± 0.021	99.10	40.580 ± 3.630	89.08
50	0.255 ± 0.021	98.90	43.132 ± 3.190	94.68

**Table 7:** Pairwise Pearson correlations among quality indicators.

Variable Pairs	r	*p*-Value	Holm-Corrected *p*-Value
Protein (dry basis) vs. POD	−0.224	0.235	0.704
Protein (dry basis) vs. SOD	−0.595	<0.001 *	0.003 *
Amylose (dry basis) vs. POD	−0.180	0.340	0.704
Amylose (dry basis) vs. SOD	−0.375	0.041 *	0.207
POD vs. SOD	0.116	0.540	0.704
Protein vs. amylose (dry basis)	0.281	0.133	0.532

* *p* < 0.05.

**Table 8:** Factors and levels used in the thin-layer paddy drying experiments.

No. of Treatments	Temperature (°C)	Relative Humidity (%)	Air Velocity (m/s)	Bed Thickness (mm)	Sample Mass (g)
30	30, 35, 40, 45, 50	20, 25, 30	3, 4	20	700

**Table 9:** Thin-layer drying models evaluated in this study.

Models	Equations	Equation No.
Newton	MR^*^ = exp(−kt)	(8)
Page	MR^*^ = exp(−kt*^n^*)	(9)
Henderson–Pabis	MR^*^ = *a*exp(−*kt*)	(10)
Logarithmic	MR^*^ = *a*exp(−*kt*) + *c*	(11)
Wang–Singh	MR^*^ = 1 + *at* + *bt*^2^	(12)
Midilli	MR^*^ = *a*exp(−*kt^n^*) + *bt*	(13)

## Data Availability

Data will be made available on request.
